# Diagnostic Value of MRI Proton Density Fat Fraction for Assessing Liver Steatosis in Chronic Viral C Hepatitis

**DOI:** 10.1155/2015/758164

**Published:** 2015-03-19

**Authors:** Francesco Paparo, Giovanni Cenderello, Matteo Revelli, Lorenzo Bacigalupo, Mariangela Rutigliani, Daniele Zefiro, Luca Cevasco, Maria Amico, Roberto Bandelloni, Giovanni Cassola, Gian Luca Forni, Gian Andrea Rollandi

**Affiliations:** ^1^Department of Radiology, E.O. Ospedali Galliera, Mura della Cappuccine 14, 16128 Genoa, Italy; ^2^Unit of Infectious Diseases, E.O. Ospedali Galliera, Mura della Cappuccine 14, 16128 Genoa, Italy; ^3^School of Radiology, University of Genoa, Via Leon Battista Alberti 4, 16132 Genoa, Italy; ^4^Unit of Pathology, E.O. Ospedali Galliera, Mura della Cappuccine 14, 16128 Genoa, Italy; ^5^Department of Medical Physics, ASL N. 5 “Spezzino”, Via XXIV Maggio 139, 19124 La Spezia, Italy; ^6^Section of Radiology, Department of Biotechnology and Legal Medicine, Policlinico Universitario “P. Giaccone”, Via del Vespro 129, 90127 Palermo, Italy; ^7^Unit of Microcitemia and Hereditary Anaemias, E.O. Ospedali Galliera, Mura della Cappuccine 14, 16128 Genoa, Italy

## Abstract

*Objective.* To assess the diagnostic performance of a T1-independent, T2^*^-corrected multiecho magnetic resonance imaging (MRI) technique for the quantification of hepatic steatosis in a cohort of patients affected by chronic viral C hepatitis, using liver biopsy as gold standard.* Methods.* Eighty-one untreated patients with chronic viral C hepatitis were prospectively enrolled. All included patients underwent MRI, transient elastography, and liver biopsy within a time interval <10 days. *Results.* Our cohort of 77 patients included 43/77 (55.8%) males and 34/77 (44.2%) females with a mean age of 51.31 ± 11.27 (18–81) years. The median MRI PDFF showed a strong correlation with the histological fat fraction (FF) (*r* = 0.754, 95% CI 0.637 to 0.836, *P* < 0.0001), and the correlation was influenced by neither the liver stiffness nor the T2^*^ decay. The median MRI PDFF result was significantly lower in the F4 subgroup (*P* < 0.05). The diagnostic accuracy of MRI PDFF evaluated by AUC-ROC analysis was 0.926 (95% CI 0.843 to 0.973) for *S* ≥ 1 and 0.929 (95% CI 0.847 to 0.975) for *S* = 2. *Conclusions.* Our MRI technique of PDFF estimation allowed discriminating with a good diagnostic accuracy between different grades of hepatic steatosis.

## 1. Introduction

It is well known that hepatitis C virus (HCV), particularly genotype 3, can lead to steatotic change in hepatocytes. In fact, the proportion of chronic hepatitis C patients with steatosis is considerable, suggesting a direct role of HCV in the intrahepatic accumulation of triglycerides, with a reported prevalence ranging from 40 to 80% [1, 2]. In addition, steatosis has been recognized as one of the factors capable of influencing both liver fibrosis progression and the rate of response to interferon-alpha-based therapy [3]. Currently, percutaneous liver biopsy remains the reference standard for the diagnosis and grading of hepatic steatosis, but its clinical application for purposes of screening, frequent monitoring, and epidemiologic studies is limited by the significant risk of bleeding, infection, and sampling error [4]. Different noninvasive imaging techniques have been proposed to assess the presence and severity of hepatic steatosis, including ultrasonography (US), computed tomography (CT), and magnetic resonance imaging (MRI) [5]. Due to its power of tissue characterization, MRI has a pivotal role for the detection and quantification of liver fat content. To this regard, the main MRI-based tools include fat-suppressed and chemical-shift water-fat separation techniques (i.e., 2- and 3-point Dixon, multiecho and multi-interference methods) and magnetic resonance spectroscopy (MRS) [6–10]. Currently, MRS is regarded as the most accurate noninvasive imaging method for assessing fatty liver, and MRS-derived fat fraction (FF) represents an objective biomarker of this condition, characterized by a strong correlation with intracellular triglyceride content [11–15]. However, MRS is not widely available, is time consuming to perform and analyze, and samples only a small portion of the liver (i.e., a volume of about 4 cm^3^) [10, 12, 15]. Due to the limitations of spectroscopy, rapid chemical-shift methods are more commonly used in the clinical practice for estimating the liver FF [8, 11, 13, 16–18]. Otherwise, the application of these ready-available MRI techniques is hindered by the presence of different confounding factors (i.e., T1 relaxation effects, T2^*^ decay, spectral complexity of fat, noise bias, B0 inhomogeneity, and eddy currents), that require proper correction [10, 12, 17–19]. More recently, in order to eliminate all major biases seen with conventional chemical shift-based methods, newer multiecho [8, 11, 13, 20] and multi-interference [10, 12, 19, 21–23] methods incorporating spectral modeling of fat have been described for the quantification of proton density fat fraction (PDFF). In addition, in chronic liver disease, hepatic steatosis may coexist with various other histological abnormalities, including fibrosis, necroinflammatory activity, and hemosiderin deposition, which may act as confounding factors on fat quantification by MRI [8]. From a clinical viewpoint, the issue regarding MRI quantification of hepatic steatosis in patients affected by chronic viral C hepatitis has been addressed in few previous works [24–26]. The purpose of our study was to assess the diagnostic performance of an original T1-independent, T2^*^-corrected multiecho MRI technique for the estimation and quantification of liver steatosis in a cohort of patients with chronic viral C hepatitis, using histology as standard of reference and assessing the influence of the other histological abnormalities on MRI PDFF measurements.

## 2. Methods

### 2.1. Inclusion of Patients

This was a prospective, monocentric, and institutional review board approved study and patient's enrollment was performed at the Unit of Infectious Diseases of our institution. From January 1st, 2013, through December 31st, 2013, 81 consecutive untreated patients with chronic viral C hepatitis were enrolled into the study after giving written informed consent. All patients were untreated (i.e., not under interferon-based therapies) at the time of enrollment. Exclusion criteria were the presence of major contraindications to 1.5T MRI (e.g., cardiac pacemaker, claustrophobia, foreign bodies, and implanted medical devices with ferromagnetic properties [27]) and/or to liver biopsy (e.g., uncorrectable coagulopathy [28]). All included patients underwent MRI, transient elastography (TE), and liver biopsy within a time interval <10 days. Severe respiratory and motion artifacts on MR images were considered as an additional post-MRI exclusion criteria to avoid unreliable measurements of MRI PDFF. After inclusion, the following laboratory values were obtained for all patients: aspartate aminotransferase (AST, expressed in IU/l), alanine transaminase (ALT, expressed in IU/L), gamma-glutamyl transpeptidase (GGT, expressed in IU/L), total bilirubin (expressed in mg/dL), platelet count (10^3^ cells per *μ*L of blood), and serum ferritin levels (expressed in ng/mL). Serum HCV-RNA levels were assessed in all patients by means of a quantitative method (real time polymerase chain reaction) and expressed in IU/mL.

### 2.2. MRI Examinations and PDFF Measurements

MRI of the liver was performed in the supine position on a 1.5T MRI scanner (Sigma HDx, General Electric Medical Systems, Milwaukee, WI, USA) using a phased array, eight-element, and flexible torso coil. All patients were carefully instructed to suspend respiration at the end of inspiration during the MRI sequence acquisition. A two-dimensional, spoiled, and multiecho gradient-echo sequence with 16 echoes was performed in the axial plane to measure hepatic PDFF. The parameters of this sequence were adjusted in order to achieve a complete correction for confounding factors such as T1 bias, T2^*^ decay, and water-fat signal interference [10, 12, 19]. To minimize T1 effects, a 20° flip angle was used at repetition time (TR) ranging from 120 to 270 msec, adjusted by the technologist to individual breath-hold capacity. To estimate water-fat signal interference and T2^*^ effects, 16 echoes were obtained at serial opposed-phase and in-phase echo times (TE) (1.1, 2.25, 3.4, 4.55, 5.7, 6.85, 8, 9.15, 10.3, 11.45, 12.6, 13.75, 14.9, 16.05, 17.2, and 18.35 msec) during a single breath hold of 12–34 seconds. Other imaging parameters were 10 mm section thickness, 0 intersection gap, 125 kHz bandwidth, one signal average, and rectangular field of view with a 128 × 96 matrix adjusted to individual body habitus and breath-hold capacity. The multiecho gradient-echo MR images were exported in DICOM format for offline postprocessing.

### 2.3. Image Interpretation and Data Analysis

All MRI datasets derived from multiecho gradient-echo images were postprocessed by a single experienced abdominal radiologist. The quantification of liver PDFF was performed with a publicly available software named C-Iron (Camelot Biomedical Systems SRL, Genoa, Italy; website: http://www.c-iron.camelotbio.com). C-Iron is a stand-alone software tool dedicated to the voxelwise measurement of T2^*^ decay for the quantification of iron overload and liver PDFF. Once acquired, the multiecho gradient-echo MR images are imported into the software. T2^*^ values and PDFFs are estimated by fitting the MRI signal (S) acquired at different TEs with the following decay model proposed by Bydder et al. [19] as follows:

(1) STE=sqrtS12exp⁡−2TET2,w∗+S22exp⁡−2TET2,f∗    +2S1S2exp⁡−TET2,w∗exp⁡−TET2,f∗cos⁡ωTE,

where *S*_1_ and *S*_2_ are the signal amplitudes of water and fat, respectively, *T*_2,w_^*^ and *T*_2,f_^*^ are the transverse relaxation times of water and fat, and *ω* = 2*π*/4.6 ms is the chemical shift between water and fat at 1.5 T. The algorithm simultaneously estimates T2^*^ and PDFF in each voxel of the image by using nonlinear least-squares fitting from all 16 echoes, assuming exponential decay and considering that fat has its own inherent T2 decay of 12 ms.

The quality of fit is assessed by means of the coefficient of determination *R* ^2^ and pixels with low-quality fit are excluded from further processing by applying appropriate thresholds on the *R* ^2^ value. The PDFF is then calculated by the following formula: FF = *S*_2_/(*S*_1_ + *S*_2_).

**Figure 1:**
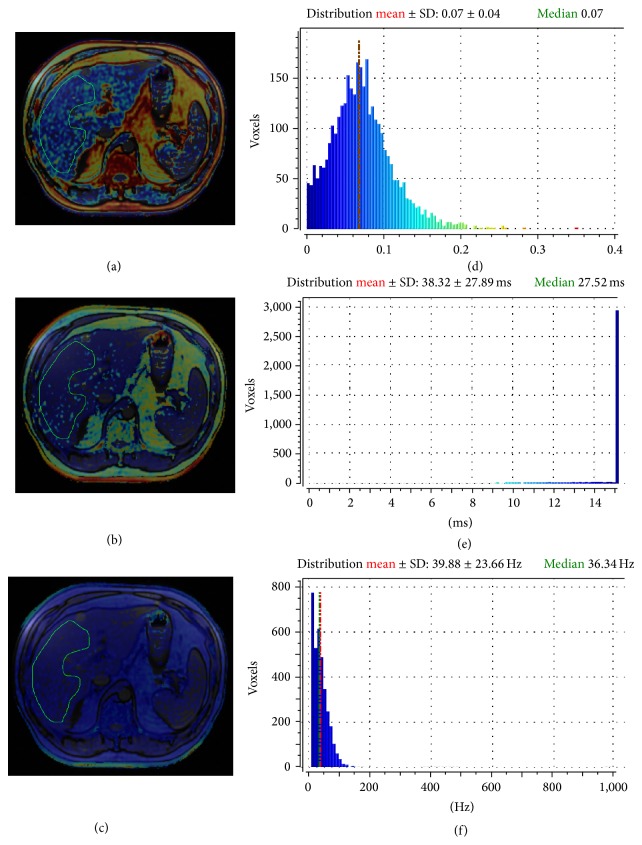
Example of ROI positioning for the calculation of MRI PDFF (a), T2^*^ (b), and R2^*^ decay (reciprocal of T2^*^, expressed in Hz) (c) in a 52-year-old male patient with chronic viral C hepatitis. The histological FF of this patient was 10%, corresponding to a steatosis grade 1 (S1). Images (d), (e), and (f) show the histogram of pixel distribution with mean values ± standard deviation and medians.

A color-coded map reflecting the estimated PDFF values in each pixel of the image is displayed and juxtaposed on the corresponding axial MRI slice. The histogram of pixel distribution with mean, median, and standard deviation of the PDFF values is computed in a freehand, elliptical, or rectangular user-adjustable ROI. A single abdominal radiologist, blind to the results of both TE and histology, performed ROI positioning. A single freehand ROI was drawn in a midhepatic axial slice including the right lobe of the liver and systematically excluding large blood vessels, biliary ducts, and focal lesions. The mean area of the ROIs was of about 40–60 cm^2^, depending on patient's anthropometric features (Figure 1). MRI PDFF and T2^*^ decay were calculated in the same ROI. Clinically significant hepatic iron overload was defined by MRI T2^*^ values less than 6.3 ms, corresponding to a liver iron concentration in dry tissue (LIC dry weight) of 4.2 mg/g [29, 30].

### 2.4. Transient Elastography

Transient elastography (TE) is a corroborate method for the assessment of liver fibrosis in patients with chronic C hepatitis. TE was performed with FibroScan (Echosens, Paris, France) with liver stiffness measurements expressed in kilopascals (values between 2.5 kPa and 75 kPa are expected) [31]. Acquisitions that do not have a correct vibration shape or a correct followup of the vibration propagation are automatically rejected by the software. Measurements of liver stiffness were performed on the right lobe of the liver through intercostal spaces in correspondence to the midaxillary line, while patients were lying in the supine position with the right arm in maximal abduction. In all included patients, TE measurements were successfully acquired (i.e., 10 correct measurements with an interquartile range lower than 30% of the median liver stiffness value [32]).

### 2.5. Liver Biopsy

Ultrasound-assisted percutaneous liver biopsy was performed with an intercostal approach using 15- to 18-gauge needles. All biopsy specimens were fixed in formalin and embedded in paraffin. A single expert liver pathologist, blind to the results of both TE and MRI, read the specimens on site. Fibrosis was semiquantitatively evaluated and staged on a 5-point scale from 0 to 4 according to the METAVIR scoring system (F0, absent; F1, enlarged fibrotic portal tract; F2, periportal or initial portal-portal septa but intact architecture; F3, architectural distortion but no obvious cirrhosis; and F4, cirrhosis) [33]. Necroinflammatory activity, represented by piecemeal necrosis and focal lobular necrosis, was semiquantitatively evaluated by using the histological activity index described in the METAVIR system and graded as follows: 0, no activity; 1, mild; 2, moderate; 3, severe [33, 34]. Liver steatosis was determined by estimating the percentage of fat-containing hepatocytes on hematoxylin-eosin stained specimens and graded according to the method of Kleiner et al. [35]: S0, steatosis in fewer than 5% of hepatocytes; S1, 5%–33% of fatty hepatocytes; S2, 34%–66%; and S3, more than 66%. We also considered the percentage of fatty hepatocytes as an absolute value which was defined as histological fat fraction. Following the clinical standard, a Perl's Prussian blue reaction was applied to detect the presence of hemosiderin granules in biopsy specimens. The following ordinal 4-point scoring system was employed: grade 0, no iron deposits; grade 1, mild; grade 2, moderate; grade 3, high iron content [36].

### 2.6. Statistical Analysis

Descriptive statistics were produced for demographic, clinical, and laboratory characteristics of patients. Categorical data were expressed as number and percentage, while continuous data were expressed as mean and standard deviation (SD) or median and range (from minimum to maximum). The normal distribution of different datasets was assessed by means of the D'Agostino-Pearson test. Nominal statistical significance was defined with a *P* of 0.05. The correlation of histological FF with MRI PDFF was tested by means of Spearman's rank test, using both the arithmetic mean and the median of MRI PDFF values. Spearman's rho (*r*) values were interpreted as follows: for values of *r* of 0.9 to 1, the correlation is very strong; for values of *r* between 0.7 and 0.89, correlation is strong; for values of *r* between 0.5 and 0.69, correlation is moderate; for values of *r* between 0.3 and 0.4.9, correlation is moderate to low; for values of *r* between 0.16 and 0.29, correlation is weak to low; for values of *r* below 0.16, correlation is too low to be meaningful. Since the median MRI PDFF showed a better correlation with the histological FF, this parameter was adopted for the subsequent statistical analysis. The correlation of median MRI PDFF values with histological FF was also tested using a partial correlation model, where liver stiffness, expressed in kPa, and T2^*^ decay, expressed in ms, were introduced as confounding covariates. The cohort of patients was further stratified according to each histological feature of the METAVIR system, including fibrosis stage (F), inflammatory activity (A), and steatosis grade (S). Box plots were used to study the distribution of MRI PDFF according to each stage of fibrosis, inflammatory activity, and steatosis, and the presence of significant differences in the median MRI PDFF values among subgroups of patients was tested using the nonparametrical Kruskal-Wallis test. After a positive Kruskal-Wallis test (*P* value < 0.05), a post-hoc analysis was conducted performing pairwise comparisons between subgroups. The diagnostic performance of MRI for detecting the correct histological grade of hepatic steatosis was assessed by using receiver operating characteristic (ROC) curves. For the ROC curve analysis, the area under curve (AUC), optimal cutoff values, sensitivity, specificity, and positive and negative predictive values were calculated. Optimal cutoff values of MRI PDFF were chosen to maximize the sum of sensitivity and specificity for two steatosis thresholds: S0 versus S1-S2 (*S* ≥ 1) and S0-S1 versus S2 (*S* = 2). Ultimately, the MRI PDFF was introduced as dependent variable in a multiple regression model, using patient's age, BMI, TE liver stiffness values, MRI T2^*^ values, METAVIR stage of fibrosis, inflammation, steatosis, and histological FF as independent variables.

## 3. Results

Four patients were excluded due to severe motion/respiratory artifacts in their MRIs, precluding an accurate measurement of PDFF. The resulting cohort of 77 patients with chronic C hepatitis included 43/77 (55.8%) males and 34/77 (44.2%) females with a mean age of 51.31 ± 11.27 (from 18 to 81) years and a mean BMI of 22.39 ± 2.27 (from 18.43 to 27). Seventy-one/77 patients (92.2%) presented detectable serum HCV-RNA levels (above the detection threshold of 15 IU/mL of our method), while 6/77 patients (7.8%) were in sustained virological response. In this latter subgroup, the standard treatment with peginterferon and ribavirin was stopped at least 18 months before the time of inclusion. Demographic, clinical, and laboratory characteristics of patients are summarized in Table 1. The mean MRI PDFF of our cohort of patients, expressed in percentage units, was 11.76 ± 4.73 with a median of 5.87 (from 0.7 to 17.01). The mean liver T2^*^ value was 30.33 ± 5.98 ms with a median of 31.32 ms (from 16.36 to 43.6 ms). We did not find patients with a histological steatosis of grade 3 (S3), and hemosiderin deposits were found in 4 patients. In addition, T2^*^ values were not indicative of hepatic iron overload of clinical significance (i.e., below the threshold of 6.3 ms) in any patient. Therefore, we were not able to assess the diagnostic performance of MRI PDFF for the detection of severe steatosis (i.e., grade *S*3, >66% fat-containing hepatocytes) and the potential confounding effect of iron overload on MRI PDFF measurements. On the other hand, we introduced T2^*^ values in the partial correlation model in order to verify their influence on the correlation between MRI PDFF and histological FF.

### 3.1. Correlation and Subgroup Analysis

**Figure 2:**
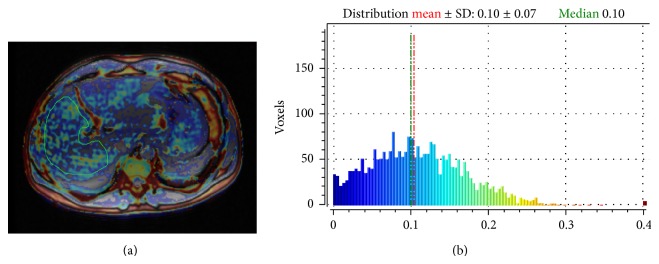
Calculation of MRI PDFF in a 45-year-old male patient with chronic viral C hepatitis (a). The median MRI PDFF value is 10% (b), while histological FF of the patient was 8%, corresponding to a steatosis grade 1 (S1).

**Figure 3:**
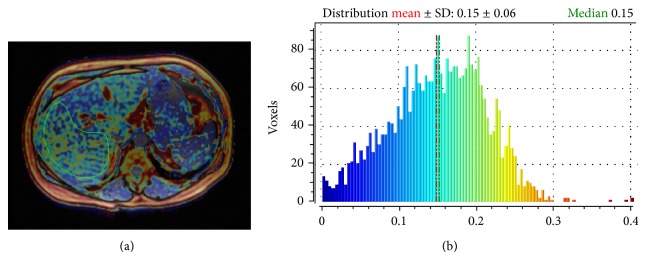
Calculation of MRI PDFF in a 45-year-old male patient with chronic viral C hepatitis (a). The median MRI PDFF value is 15% (b), while histological FF of the patient was 37%, corresponding to steatosis grade 2 (S2).

**Figure 4:**
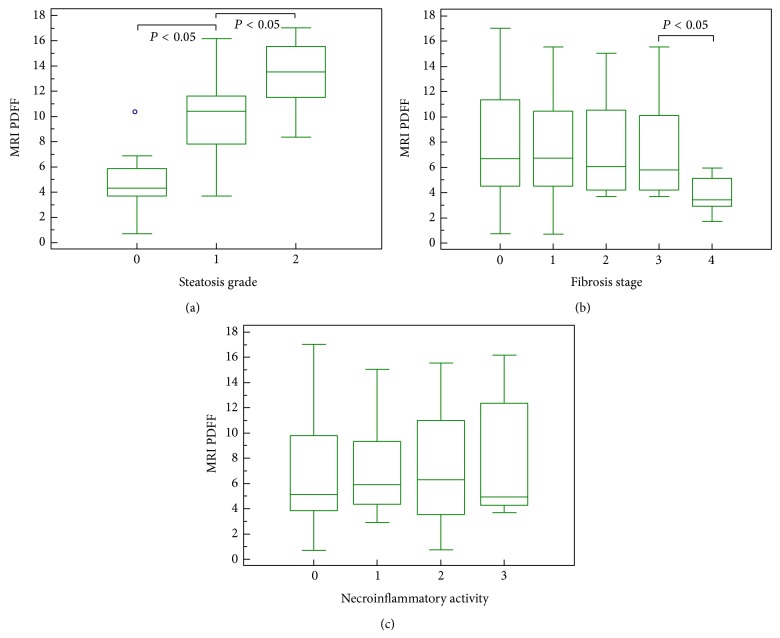
Box-and-whisker plots for MRI PDFF measurements in relation to each grade of steatosis (a), fibrosis (b), and necroinflammatory activity (c). The top and the bottom of the boxes are the first and third quartiles, respectively. The length of the box represents the interquartile range including 50% of the values. The line through the middle of each box represents the median. The error shows the minimum and maximum values (range). An outside value (separate point) is defined as a value that is smaller than the lower quartile minus 1.5 times the interquartile range or larger than the upper quartile plus 1.5 times the interquartile range.

The correlation of the mean MRI PDFF value with the histological FF was moderate (*r* = 0.624, 95% CI for rho 0.465 to 0.744, *P* < 0.0001), while the correlation of the median MRI PDFF value with the histological FF was strong (*r* = 0.754, 95% CI for rho 0.637 to 0.836, *P* < 0.0001). The median MRI PDFF values for each steatosis grade were: 4.3 (0.7–10.09) for S0; 10.4 (3.7–16.2) for S1; 13.5 (8.4–17.01) for S2 (*P* < 0.05) (Figures 2 and 3). Stratifying the cohort of patients according to the METAVIR stages of parenchymal fibrosis, the median MRI PDFF values resulted in significantly different among different subgroups (*P* < 0.05 with the Kruskal-Wallis test). The post-hoc analysis showed that the median MRI PDFF in the F4 subgroup was significantly lower than in the other subgroups of patients (*P* < 0.05) (Table 2). Stratifying the cohort of patients according to the METAVIR stages of necroinflammatory activity, the Kruskal-Wallis test did not reveal a significant difference among the median MRI PDFF values of the four subgroups of patients (*P* > 0.05) (Figure 4). Box-and-whisker plots for MRI PDFF measurements in relation to each grade of steatosis, fibrosis, and necroinflammatory activity are shown in Figure 4.

### 3.2. Diagnostic Accuracy of MRI PDFF

**Figure 5:**
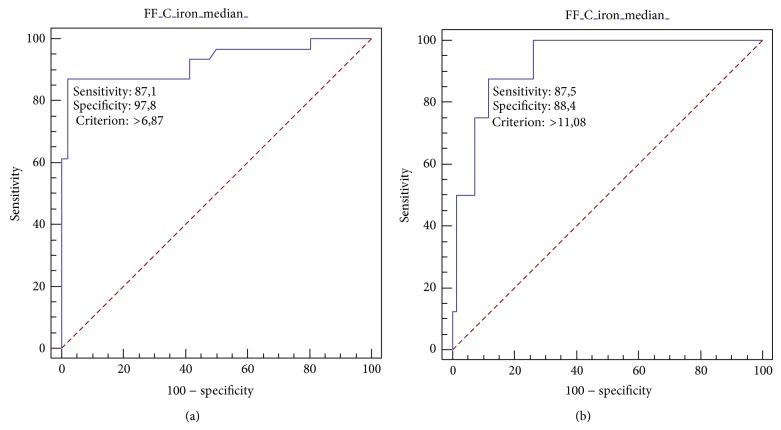
ROC curve analysis of MRI PDFF for patients with steatosis *S* ≥ 1 (S0 versus S1-S2). The area under the ROC curve is 0.926 (95% CI 0.74–0.94) (a). ROC curve analysis of MRI PDFF for patients with steatosis *S* = 2 (S0-S1 versus S2). The area under the ROC curve is 0.929 (95% CI 0.806 to 0.968) (b).

The diagnostic accuracy of MRI PDFF evaluated by AUC-ROC analysis was 0.926 (standard error 0.0354, 95% CI 0.843 to 0.973) for *S* ≥ 1 and 0.929 (standard error 0.0363, 95% CI 0.847 to 0.975) for *S* = 2. The best MRI PDFF cutoff value to differentiate between S0 and S1-S2 patients was 6.87, showing a sensitivity of 87.10% (95% CI 70.2–96.4), a specificity of 97.83 (95% CI 88.5–99.9%), a positive predictive value (PPV) of 96.4% (95% CI 81.7–99.9), and a negative predictive value (NPV) of 91.8% (95% CI 80.4–97.7) (Figure 5(a)). The best MRI PDFF cutoff value to differentiate between S0-S1 and S2 patients was 11.08, showing a sensitivity of 87.5% (95% CI 47.3–99.7), a specificity of 88.41% (95% CI 78.4–94.9), a positive predictive value (PPV) of 46.7% (95% CI 20.5–74.3), and a negative predictive value (NPV) of 98.4% (95% CI 91.3–100) (Figure 5(b)).

### 3.3. Influence of Confounding Variables on MRI PDFF Measurements

The correlation between MRI PDFF and histological FF was strong even in a partial correlation model, using TE liver stiffness values (expressed in kPa) and T2^*^ decay (expressed in ms) as covariates (*r* = 0.775, *P* < 0.0001).

The multiple regression analysis showed that only steatosis grade at histology and histological FF were factors independently associated to the median MRI PDFF (Table 3).

## 4. Discussion

Liver biopsy with histological visualization of hepatocellular fat vacuoles remains the reference method in order to determine the grade of steatosis in chronic liver diseases, but it is an invasive procedure, which can study only a small portion of the liver (i.e., 1/50,000 of the total volume) [4, 30]. Discomfort and bleeding are well-known procedure-related complications. In addition to sampling errors, routine histological examination is semiquantitative and observer-dependent, and grading is performed with broad severity brackets [37]. Therefore, a noninvasive and objective assessment on a continuous scale may be preferable to biopsy in both clinical practice and research. Different noninvasive imaging methods, including US, CT, and MRI, have been employed to provide an estimate of liver steatosis. It causes reduced liver attenuation at CT, resulting in low hepatic density compared to spleen during precontrast and portal venous phase imaging [5]. Despite the development of quantitative methods of image analysis to assess the severity of hepatic steatosis with CT [5], the clinical implementation of this imaging modality is hampered by exposure to ionizing radiation, which limits its application for repeated measurements in monitoring disease progression [9, 15]. Using B-mode US imaging, an indirect estimate of hepatic steatosis is obtained by comparing the echogenicity of the liver parenchyma with that of the cortex of the right kidney. This comparison may be performed in either semiquantitative (i.e., normal liver echotexture, minimal, mild, moderate, and severe hyperechogenicity [5]) or quantitative modality (i.e., hepatorenal index [38]). Hepatorenal index calculation has been presented as an effective tool for differentiating patients with steatosis from those without steatosis [38], showing a strong correlation with the histological FF (*r* = 0.71, *P* < 0.0001). However, it has to be kept in mind that a high echogenicity of the liver parenchyma in not synonymous of steatosis. In fact, this appearance of the liver at B-mode US may also be related to the presence of parenchymal fibrosis and liver iron overload, leading to overestimation of the true steatosis grade or misdiagnosis.

MRI-based techniques have been widely employed to determine the presence and grade the severity of hepatic steatosis, and MRS is regarded as the most accurate noninvasive method for assessing this condition [11–14]. In fact, FF calculated from spectroscopy-determined proton densities has shown a strong direct correlation with the intracellular triglyceride content [14, 15]. However, this expensive and time-consuming technique is not widely available and is mainly limited to research settings. Advanced multiecho and multiinterference MRI techniques allow measurement of PDFFs that are corrected for confounding factors, including B0 inhomogeneity, T1 bias, T2^*^ decay, and multifrequency signal interference effects caused by protons in fat [10–13, 17–19]. The most recent studies are giving encouraging results on clinical grounds, demonstrating a strong correlation between MRI PDFF and hepatic steatosis grade determined by histological validation, and proposing MRI PDFF as a valid noninvasive biomarker for assessing liver fat content [21, 23]. Idilman et al. have recently shown that sequential MRI PDFF quantification may also be employed for monitoring the longitudinal changes of the liver fat content in NAFLD patients [23]. In our work, we performed the quantification of MRI PDFF by means of a comprehensive model derived from that proposed by Bydder et al. [19], incorporating correction for T1- and T2^*^ relaxation effects, B0 inhomogeneity, and spectral complexity of fat. This method of analysis has never been employed in a homogeneous cohort of patients with chronic C hepatitis. The prevalence of steatosis in chronic C hepatitis is about 40%, which represents an approximately 2-fold increase compared to the prevalence of steatosis in chronic B hepatitis (i.e., 20%) [1]. In fact, HCV infection is considered to be directly involved in the accumulation of triglycerides in hepatocytes (the so-called “viral” steatosis) [39]. According to the literature, we found in our cohort of patients a prevalence of steatosis of 40.26%. In HCV-related steatosis, the percentage of fat-containing hepatocytes is usually mild to moderate (i.e., 10–20%) [34], as it was observed in our study, with a median histological FF in patients with relevant steatosis (*S* ≥ 1) of 15%. In addition, we observed a lack of patients with grade 3 steatosis (i.e., >66% of fat-containing hepatocytes). The severity of steatosis seems to correlate with the level of HCV replication (i.e., HCV RNA levels in serum) [3], and it significantly reduces or disappears when patients are successfully treated with antivirals [40]. Interestingly, and according to our results, as the liver disease progresses to cirrhosis (i.e., F4 METAVIR stage of fibrosis), there is a trend of reduction of parenchymal steatosis [41], a phenomenon already observed in NAFLD [42]. Some longitudinal studies underscored the role of steatosis in fibrosis progression. In a recent study on paired liver biopsies performed in 135 untreated patients with chronic C hepatitis [43], steatosis was the only independent factor predictive of fibrosis progression. The progression of fibrosis was significantly related to the percentage of hepatocytes with steatosis [43]. Given the clinical importance of steatosis detection and grading in chronic viral C hepatitis, we aimed to assess the clinical value of MRI PDFF as a noninvasive biomarker of fatty liver, finding a significant, strong correlation of the MRI PDFF with the histological FF. According to the results of Tang et al. [10], we noticed that MRI PDFF values are lower than histological figures, and MRI PDFF cutoff values to distinguish between different steatosis grades are not comparable with the histological ones. This is not surprising, since histologic examination assesses the percentage of fat-containing cells in the biopsy specimens and does not measure the volumetric fat content in a wide portion of liver parenchyma. With MRI PDFF, the proportion of mobile protons contained within fat molecules of three-dimensional liver voxels is quantified [8, 10, 12]. Therefore, MRI PDFF and histological FF assess different aspects of steatosis.

Our study has some limitations. As mentioned above, the lack of patients with a grade 3 steatosis may be considered an intrinsic limitation when examining a cohort of patients affected by chronic viral C hepatitis. Therefore, we were not able to assess the diagnostic performance of PDFF for discriminating between S0-S2 and S3 patients. In addition, we did not find cases of clinically significant MRI-detectable iron overload (i.e., MRI T2^*^ values <6.3 ms [29]), and the presence of hemosiderin deposits was appreciable in only few cases. This may be due to the low number of cirrhotic patients in our cohort; in fact, it is known that histologically detectable iron is more frequently associated with advanced parenchymal fibrosis and cirrhosis [44]. Therefore, we were not able to reliably assess the influence of hepatic iron accumulation on the MRI PDFF measurements. Nevertheless, we decided to introduce T2^*^ decay as a confounding covariate in the partial correlation model, finding that its influence on the correlation between MRI PDFF and histological FF was not significant. A point of strength of our study is that we kept a reasonably low time-interval between MRI, liver biopsy, and TE (<10 days), thus avoiding any meaningful change in the hepatic fat content during the biopsy-MRI imaging interim. In addition, we performed a double check of the influence of parenchymal fibrosis on MRI PDFF measurements, introducing TE values of liver stiffness in the partial correlation model and both TE values and METAVIR stage of fibrosis in the multiple linear regression analysis.

## 5. Conclusions

MRI PDFF is a promising technique for the noninvasive assessment of liver steatosis in patients with chronic viral C hepatitis. In particular, MRI PDFF has shown a strong correlation with the histological FF, and this correlation seems to be influenced by neither the stage of parenchymal fibrosis nor the necroinflammatory activity. In addition, MRI PDFF allows discrimination between different histological grades of steatosis with good diagnostic accuracy. Further studies on larger cohort of patients involving adequate control groups are needed to get a complete clinical validation of this technique in patients with chronic viral C hepatitis.

## Figures and Tables

**Table 1:** Characteristics of patients and results of histological analysis of liver biopsy specimens.

Characteristics of patients	Proportions, means ± standard deviation	Percentages, medians, and range
Males	43/77	55.8%
Females	34/77	44.2%
Age	51.31 ± 11.27	51 (18–81)
BMI	22.39 ± 2.27	23 (18.43–27)
Serum AST level (U/L)	66.49 ± 65.93	48 (18–293)
Serum ALT level (U/L)	62.83 ± 53.13	51 (15–302)
Serum GGT level (U/L)	92.63 ± 90.92	62 (11–368)
Total bilirubin (mg/dL)	1.05 ± 1.26	0.7 (0.2–9)
Platelet count (10^3^ cells/*µ*L)	196.25 ± 62.06	199 (99–462)
Serum ferritin level (ng/mL)	167.43 ± 141.68	134.3 (13.3–700.4)
HCV-RNA (IU/mL)	1.96 × 10^6^ ± 1.91 × 10^6^	1.34 × 10^6^ (2.99 × 10^3^–6.65 × 10^6^)
Stiffness (kPa)	12.86 ± 11.57	7.2 (3.8–55)

Histology		
Histological fat fraction	9.09 ± 12.68	3 (0–45)

Steatosis grade (S)		
Grade 0 (<5%)	46/77	59.7%
Grade 1 (5–33%)	23/77	29.9%
Grade 2 (33–66%)	8/77	10.4%
Grade 3 (>66%)	0/77	0%

Necroinflammation (A)		
Grade 0	25/77	32.5%
Grade 1	33/77	42.8%
Grade 2	14/77	18.2%
Grade 3	5/77	6.5%

Fibrosis (F)		
F0 (none)	23/77	29.9%
F1 (perisinusoidal or periportal)	14/77	18.2%
F2 (perisinusoidal and portal/periportal)	12/77	15.5%
F3 (bridging fibrosis)	18/77	23.4%
F4 (cirrhosis)	10/77	13%

Histologically detectable iron		
Grade 0	73/77	94.8%
Grade 1	2/77	2.6%
Grade 2	2/77	2.6%
Grade 3	0/77	0%

Values are expressed as percentages, means ± standard deviation, and medians (min–max).

Legend: BMI, body mass index.

**Table 2:** Distribution of MRI PDFF values according to different METAVIR stages of hepatic fibrosis. The Kruskal-Wallis test revealed a significant difference between groups (*P* < 0.05). The post-hoc analysis demonstrates that the median MRI PDFF value of the F4 subgroup is significantly lower than that of the other subgroups of patients.

Post-hoc analysis: distribution of PDFF according to METAVIR stages of fibrosis				
Factor	*n*	Median (range)	Average rank	Pairwise comparisons with a significant result (*P* < 0.05)
F0	23	6.7 (0.72–17.01)	44.61	F0 versus F4
F1	14	6.7 (0.7–15.54)	43.25	F1 versus F4
F2	12	6.07 (3.68–15.04)	40.33	F2 versus F4
F3	18	5.78 (3.7–15.54)	39.36	F3 versus F4
F4	10	3.43 (1.72–5.95)	17.90	F4 versus F0/F1/F2/F3

**Table 3:** Multiple regression analysis. MRI PDFF is the dependent variable of the model. Histological FF and the histological grade of steatosis were the only two factors independently and significantly correlated to MRI PDFF. *P* values below the level of statistical significance (*P* < 0.05) are marked with the asterisk.

Regression equation					
Independent variables	Coefficient	Standard error	*r*_partial_	*t*	*P*
(Constant)	8.3980				
Age	−0.01905	0.02438	−0.09435	−0.781	0.4372
BMI	−0.1480	0.1248	−0.1424	−1.186	0.2397
Necroinflammation (A)	0.07004	0.3184	0.02667	0.220	0.8266
Fibrosis (F)	−0.5041	0.3546	−0.1699	−1.422	0.1596
Steatosis (S)	2.3698	1.1144	0.2497	2.127	0.0371^*^
Liver stiffness	0.01464	0.04543	0.03903	0.322	0.7483
Histological FF	0.1325	0.05975	0.2597	2.218	0.0299^*^
T2^*^	0.03749	0.04626	0.09781	0.810	0.4205
